# Editorial: Genetics, Evolution, and Conservation of Neotropical Fishes

**DOI:** 10.3389/fgene.2019.01124

**Published:** 2019-11-04

**Authors:** Rodrigo Augusto Torres, Roberto Ferreira Artoni

**Affiliations:** ^1^Laboratório de Genômica Evolutiva e Ambiental, Departamento de Zoologia, Centro de Biociências, Universidade Federal de Pernambuco, Recife, Brazil; ^2^Laboratório de Genética e Evolução, Departamento de Biologia Estrutural, Molecular e Genética, Universidade Estadual de Ponta Grossa, Ponta Grossa, Brazil

**Keywords:** Chromosome, molecular marker, Genetic population, Evolutionary, fish

Fishes are a more basal and speciose vertebrate group and they occupy the most diverse aquatic environments on earth. Fish are extremely diverse, especially in biologically mega diverse countries such as those in the Neotropical region, encompassing a very large area in the Americas. The amazing diversity alone generates great interest in fish biology, ecology and evolution, but despite that interest, many challenges remain. Today, new genetic methods are available to study evolutionary and ecological processes that have influenced and given rise to their wide diversity and the many habitats that these fish occupy. Consequently, these genetic methods can also serve as tools for better conservation and management. Today, we must ask ourselves what are the classic questions not yet answered? What new information is available and useful to answer those questions? Where will this new science lead us into the future? To begin answering those questions, we asked these, and other, questions of the scientific community with the goal of gathering together answers to those questions for the Frontiers in Genetics, which celebrates the 30th anniversary of the first Symposium on Cytogenetics and Fish Genetics.

Traditionally, karyotpye analysis is among the genetic methods most often applied to Neotropical fish. Since the 1970s, karyotypes studies have been used to describe diversity and thereby provide support for evolutionary systematics, taxonomy and the evolution of karyotypes themselves. Banding and classical chromosomal markers, now used with the fluorescence *in-situ* hybridization (FISH) method and its derivatives, have advanced Neotropical fish cytogenetics which has allowed for more extensive comparative analyses with African fish (Carvalho et al.) and to investigate incipient speciation (Ferreira et al.) and evolutionary divergences of sex chromosomes (Sember et al.), as well as complex chromosomal rearrangements, that are a recurrent feature found in many neotropical fish families (Machado et al.). With integration of cytogenetics and molecular markers, a new cycle of methodological developments has come to pass, especially with the inclusion of dates that became available through massive sequencing of DNA and RNA. Trends in karyotype evolution have been refined in several species complexes, including the genera *Astyanax* (Pazza et al.) and *Mugil* (Nirchio et al.), as well as permitting the uncovering of surprising and previously unknown diversity in the armored catfish *Ancistrus* (Prizon et al.). While a good beginning, bioinformatics use in the selection of satellite DNA markers and their chromosome localization is still growing (Utsunomia et al.). This tremendous growth of information and the availability of more robust methodological tools, and finding undiscovered diversity, have resulted in a growing concern for understanding the evolutionary history of this diversity as well as its conservation (Moritz, 2002). Due to rapidly declining biodiversity of Neotropical fish, integrating conservation genetics with data describing systematics and ecological information including details of the adaptive landscape, together will become important for future research. Recently, the application of genetic evolutionary analysis in fish conservation has shown its importance by demonstrating that commercially exploited species are losing genetic variability (Allendorf et al., 2014; Pinsky and Palumbi, 2014). Investigating taxonomic uncertainties and genetic diversity (evolutionary potential) of the many species of fish, diagnosing patterns of gene flow, and the processes that structure and fragment populations are among the most important topics for the conservation of fish in their natural environments. In this volume, we bring attention to analyses of genetic structure, population diversity, and research in evolutionary history. Conservation issues include problems that arise from hydroelectric dams that lead to the disappearance of rheophilic fish fauna that previously occupied the river being dammed (Hrbek et al.). This has resulted in reduced genetic diversity and changing population structure of geographical variants in the catfishes *Steindachneridion scriptum* (Paixão et al.) and *S. parahybae*, both of which are endangered (ICMBio/MMA, 2018) (Fonseca et al.). Additionally, dams are detrimental for the pirarucu, *Arapaima gigas*, cited by the European Union that set limits on the exploitation of vulnerable or potentially endangered species (CITES, 2017) (Vitorino et al.).

The current distribution of neotropical fish genetic lineages may reflect ancient geological events such as paleo-drains, or more recent phenomena such as stream head capture.A special south Brazil hydrological system (Pampa) resulted from a long history of tectonism, climate, and sea-level changes directly affecting the diversification of fish evolutionary lineages in this biome (Ramos-Fregonezi et al.). Migratory behavior and gene flow in *Prochilodus* show little genetic divergence, however it is possible to delimit mitochondrial lineages suggestive of distinct species (Melo et al.). Natural populations of *Colossoma macropomum* comprise a single and large panmitic population in the main channel of the Solimões-Amazonas River basin, and structured populations in the headwaters of the tributaries with the greatest genetic differentiation in relation the Bolivian sub-basin (da Conceição Freitas Santos et al.). In paleo-drainage reconstructions, two putative paleo-rivers in southeastern Brazil include threatened and endemic species (Lima et al.). Pleistocene climate changes were major historical events that had a lasting impact on South American biodiversity for the endangered *Salminus brasiliensis* (ICMBio/MMA, 2018), which is found in the Pantanal basin, in which there was a period of significant population expansion (de Carvalho Mondin et al.). The use of microsatellite markers has expanded conservation tools for endemic species and populations (Souza-Shibatta et al.), and when used in conjunction with markers for phylogenetic and phylogeographic analyses, can uncover sometimes complex, often alarming, scenarios (Silva-Santos et al.). Using microsatellites, derived from transcriptome sequencing, can also be important for genetic management of natural or captive-bred commercial stocks (Jorge et al.; Ariede et al.). New strands of research constantly expand the set of evolutionary inferences about Neotropical fish.Forensic genetics has also benefitted from new tools, and using DNA barcoding has assisted in the identification of meats from different shark species (some endangered) in the domestic market in Brazil (Almerón-Souza et al.). Sequencing of cytochrome oxidase subunit 1 (COI) has helped unravel trophic relationships by the molecular identification of the stomach contents of parasitic catfishes (Bonato et al.). DNA barcoding has also revealed species complexes, such as *Megaleporinus* (Ramirez et al.) and *Schizolecis guntheri* (Souza et al.), and is a promising tool for future studies of the taxonomy of Neotropical fish. New methodologies and applications for the study of Neotropical fish genetics are constantly being developed and applied in new ways. The chromosome set manipulation by polyploidization has proved to be an important tool in obtaining sterile individuals. Recently, a protocol was developed for using flow cytometry in the confirmation of triploidy in *Astyanax altiparanae* (Xavier et al.). Beyond doubt, the coming years will be stimulated by the popularization of massive sequencing of DNA and RNA and the development of user-friendly software for analysing large data sets will follow. Improved experimental design and sequencing strategies will be important and one-such strategy for the use of next-gen sequencing technology to genotype many individuals in parallel is described for Neotropical fish (Pimentel et al.). The integration of Omics information (genome, transcriptome, and proteome) with environmental data in the analysis of biological functions and epigenetic responses is an exciting development for understanding the evolutionary biology of Neotropical fish. For example, the typical Amazonian condition, where rivers with blackwater, clearwater, and whitewater converge, transcriptomic analysis indicated phenotypic plasticity in *Triportheus albus* that allows it to survive in all these environments (Araújo et al.). In this volume, the expression of reference genes was determined by qRT-PCR to set environmental analysis parameters for study of *Odontesthes humensis* (Silveira et al.). Stable genes (i.e., good as control) were distinguished from genes that are tissue specific and unstable in that study. Another recent advance is combining physiological and gene expression analyses to better understand the ecology of Neotropical fishes. Salinity tolerances in the marine *Odonthestes* species demonstrated different physiological adaptations resulting from evolutionary processes that occurred in oceanic and estuarine environments (Silveira et al.). MicroRNA use is still incipient, but the role of these molecules in the regulation of gene expression is being investigated in fishes (Herkenhoff et al.).

Here, we certainly have not described the universe of evolutionary biology of, or the tools with which to study, Neotropical fish. Yet, we hope that those interested in this area of biological research now have an overview of the state of the art and that they will be encouraged to take additional steps to extend our current understanding of the evolutionary biology, ecology and conservation of Neotropical fish.

We greatly appreciate the invaluable contribution of all reviewers as well as the efforts of Chief Editor Dr. Samuel A. Cushman of Frontiers in Genetics: Evolutionary and Population Genetics with Research Topic and the experts and support staff at Frontiers.

## Author Contributions

RA and RT contributed equally to the preparation of this editorial manuscript of the special volume edited by these guest editors.

## Conflict of Interest

The authors declare that the research was conducted in the absence of any commercial or financial relationships that could be construed as a potential conflict of interest.
